# Supplementary material for: Clinical and Microbiological Efficacy of Pyrophosphate Containing Toothpaste: A Double-Blinded Placebo-Controlled Randomized Clinical Trial
Source: Microorganisms. 2020 Nov 17;8(11):1806. doi: 10.3390/microorganisms8111806 (PMC7698517; doi:10.3390/microorganisms8111806)
Supplement: Supplementary file 1 [file microorganisms-08-01806-s001.pdf]

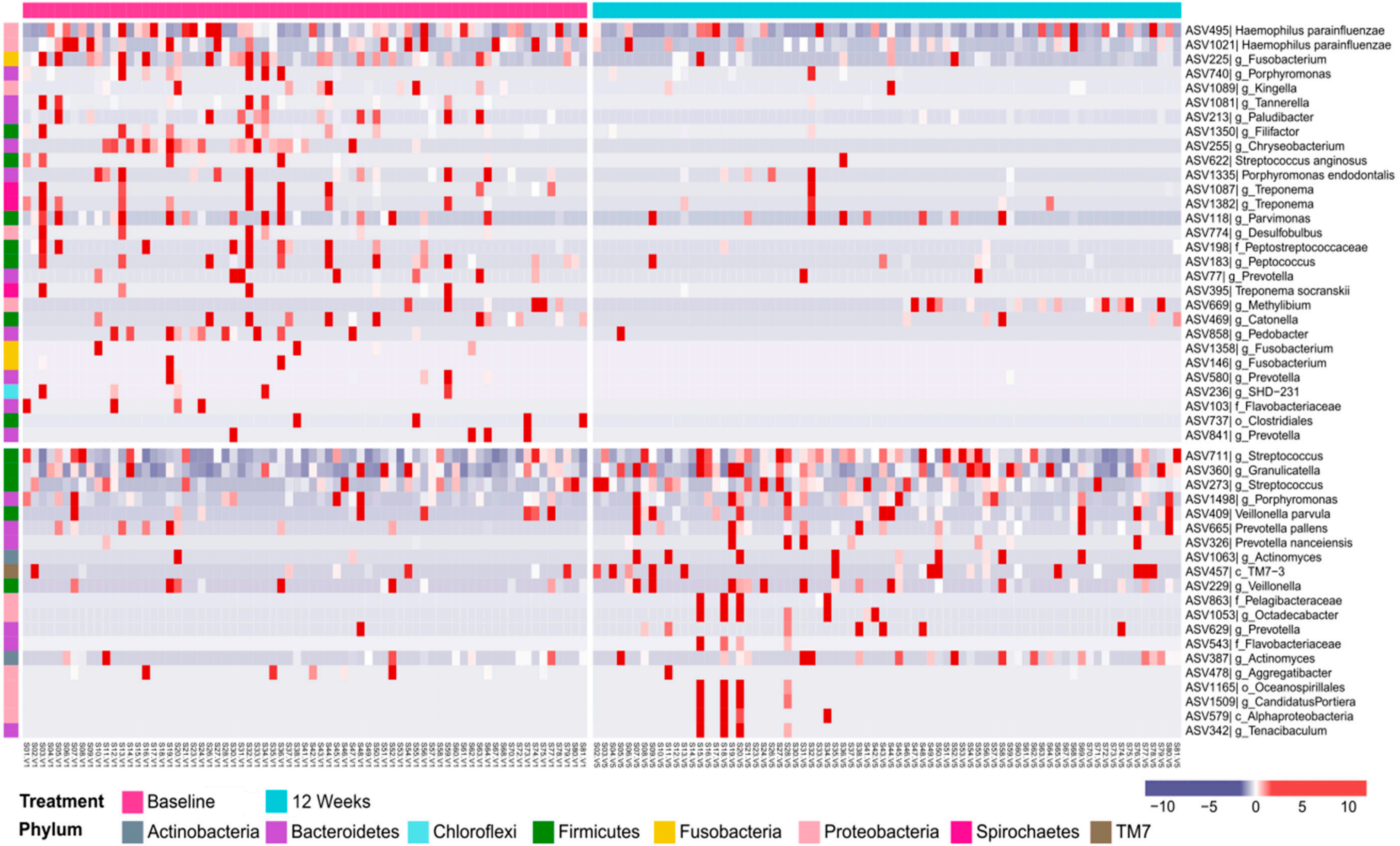

Figure S1. Linear discriminant analysis effect sized (LEfSE) analysis of microbiome in baseline and 12 weeks.

Table S1. Species which showed significant difference (linear discriminant analysis (LDA) score ≥ 2.5) in linear discriminant analysis effect sized (LEfSe) analysis. (A) ASVs of control group which showed significant difference between baseline and 12 weeks. (B) ASVs of test group which showed significant difference between baseline and 12 weeks. u.i, Unidentified

(A) Control

Phylum	Class	Order	Family	Genus	Species	ASV	Time	LDA score	P value	Mean RA in baseline (%)	Mean RA in 12 weeks (%)
Bacteroidetes	Flavobacteriia	Flavobacteriales	Flavobacteriaceae	Capnocytophaga	u.i	ASV110	Baseline	2.4569	0.018	0.05±0.18	0
Fusobacteria	Fusobacteriia	Fusobacteriales	Fusobacteriaceae	Fusobacterium	u.i	ASV225	Baseline	3.0469	0.0047	0.25±0.40	0.06±0.13
Bacteroidetes	Flavobacteriia	Flavobacteriales	Weeksellaceae	Chryseobacteriu m	u.i	ASV255	Baseline	2.6588	2.25E-05	0.07±0.15	0
Bacteroidetes	Bacteroidia	Bacteroidales	Porphyromonadaceae	Paludibacter	u.i	ASV213	Baseline	2.3868	0.034	0.04±0.13	0.0024±0.01

(B) Test

Phylum	Class	Order	Family	Genus	Species	ASV	Time	LDA score	P value	Mean RA in baseline (%)	Mean RA in 12 weeks (%)
Proteobacteria	Gammaproteobacteria	Pasteurellales	Pasteurellaceae	Haemophilus	parainfluenzae	ASV495	Baseline	4.1743	3.55E-05	4.72±5.14	1.57±3.25
Proteobacteria	Betaproteobacteria	Neisseriales	Neisseriaceae			ASV309	Baseline	3.4063	0.034	0.03±0.14	0.0007±0.0042
Proteobacteria	Betaproteobacteria	Neisseriales	Neisseriaceae			ASV354	Baseline	3.341	0.0093	0.45±1.11	0.09±0.42
Fusobacteria	Fusobacteriia	Fusobacteriales	Fusobacteriaceae	Fusobacterium	u.i	ASV225	Baseline	3.311	0.015	0.57±1.10	0.14±0.47
Proteobacteria	Gammaproteobacteria	Pasteurellales	Pasteurellaceae	Aggregatibacter		ASV675	Baseline	3.2736	0.045	0.37±1.51	0.0067±0.04
Bacteroidetes	Bacteroidia	Bacteroidales	Porphyromonadaceae	Tannerella	u.i	ASV1081	Baseline	3.2389	0.0016	0.30±1.11	0.0048±0.03
Proteobacteria	Gammaproteobacteria	Pasteurellales	Pasteurellaceae	Haemophilus	parainfluenzae	ASV1021	Baseline	3.2309	0.011	0.54±1.12	0.20±0.57

Proteobacteria	Betaproteobacteria	Burkholderiales	Comamonadaceae			ASV570	Baseline	3.2246	0.020	0.70±1.06	0.37±0.70
Firmicutes	Clostridia	Clostridiales	Lachnospiraceae	Catonella	u.i	ASV469	Baseline	3.178	0.024	0.015±0.043	0.0013±0.0048
Firmicutes	Clostridia	Clostridiales	Lachnospiraceae			ASV479	Baseline	3.0772	0.027	0.028±0.14	0.002±0.014
Chloroflexi	Anaerolineae	Anaerolineales	Anaerolinaceae	SHD-231	u.i	ASV236	Baseline	3.0389	0.039	0.016±0.07	0
Bacteroidetes	Flavobacteriia	Flavobacteriales	Flavobacteriaceae	Capnocytophaga	u.i	ASV900	Baseline	3.0351	0.050	0.53±0.80	0.47±1.46
Bacteroidetes	Bacteroidia	Bacteroidales	Prevotellaceae	Prevotella	u.i	ASV594	Baseline	2.952	0.0052	0.03±0.066	0
Firmicutes	Clostridia	Clostridiales	Peptostreptococcaceae	Filifactor	u.i	ASV1350	Baseline	2.9113	0.023	0.12±0.48	0.004±0.016
Spirochaetes	Spirochaetes	Spirochaetales	Spirochaetaceae	Treponema	u.i	ASV787	Baseline	2.6795	0.039	0.053±0.22	0

Bacteroidetes	Flavobacteriia	Flavobacteriales	Weeksellaceae	Chryseobacterium	u.i	ASV255	Baseline	2.6479	0.0013	0.052±0.19	0
Proteobacteria	Deltaproteobacteria	Desulfobacterales	Desulfobulbaceae	Desulfobulbus	u.i	ASV774	Baseline	2.5715	0.020	0.045±0.17	0
Firmicutes	Bacilli	Lactobacillales	Streptococcaceae	Streptococcus		ASV711	12 Weeks	4.3652	0.0017	4.28±4.65	9.06±10.15
Proteobacteria	Gammaproteobacteria	Thiotrichales	Thiotrichaceae	Cocleimonas	u.i	ASV868	12 Weeks	3.6995	0.040	0.25±1.19	1.29±3.55
Firmicutes	Bacilli	Lactobacillales	Carnobacteriaceae	Granulicatella	u.i	ASV360	12 Weeks	3.496	0.0031	0.57±0.53	1.17±1.08
Bacteroidetes	Bacteroidia	Bacteroidales	Prevotellaceae	Prevotella	u.i	ASV817	12 Weeks	3.4407	0.023	0	0.0074±0.023
Firmicutes	Bacilli	Lactobacillales	Aerococcaceae	Abiotrophia	u.i	ASV648	12 Weeks	3.2191	0.038	0.082±0.23	0.39±0.86
Firmicutes	Clostridia	Clostridiales	Mogibacteriaceae	unidentified	u.i	ASV882	12 Weeks	3.1272	0.033	0.0084±0.042	0.020±0.070

Actinobacteria	Actinobacteria	Actinomycetales	Actinomycetaceae	Actinomyces	u.i	ASV387	12 Weeks	3.0968	0.006	0.0026±0.007	0.015±0.034
Bacteroidetes	Flavobacteriia	Flavobacteriales	Flavobacteriaceae	Olleya	u.i	ASV714	12 Weeks	3.0838	0.044	0.045±0.25	0.27±0.81
Proteobacteria	Alphaproteobacteria	Rhodobacterales	Rhodobacteraceae	Octadecabacter	u.i	ASV1053	12 Weeks	2.8794	0.044	0	0.021±0.068
Bacteroidetes	Bacteroidia	Bacteroidales	Prevotellaceae	Prevotella	nanceiensis	ASV326	12 Weeks	2.8573	0.024	0.0001±0.0007	0.041±0.17
Bacteroidetes	Saprospirae	Saprospirales	Saprospiraceae	unidentified	u.i	ASV1500	12 Weeks	2.7901	0.041	0.037±0.19	0.14±0.37
Proteobacteria	Betaproteobacteria	Neisseriales	Neisseriaceae	Neisseria		ASV1433	12 Weeks	2.7521	0.043	0.0134±0.042	0.094±0.20
Proteobacteria	Gammaproteobacteria	Thiotrichales	Thiotrichaceae	Leucothrix	u.i	ASV784	12 Weeks	2.7025	0.021	0.023±0.11	0.098±0.29
